# Socioeconomic inequalities are still a barrier to full child vaccine coverage in the Brazilian Amazon: a cross-sectional study in Assis Brasil, Acre, Brazil

**DOI:** 10.1186/s12939-014-0118-y

**Published:** 2014-11-27

**Authors:** Fernando Luiz Cunha Castelo Branco, Thasciany Moraes Pereira, Breno Matos Delfino, Athos Muniz Braña, Humberto Oliart-Guzmán, Saulo Augusto Silva Mantovani, Antonio Camargo Martins, Cristieli Sérgio de Menezes Oliveira, Alanderson Alves Ramalho, Claudia Torres Codeço, Mônica da Silva-Nunes

**Affiliations:** Universidade Federal do Acre, BR 363 km 04, 69919-769 Rio Branco, Acre Brazil; Scientific Computing Program, Oswaldo Cruz Foundation, Avenida Brasil, 4365 Rio de Janeiro, RJ Brazil

**Keywords:** Vaccine, Coverage, Child, Amazon, Cobertura vacinal, Saúde da criança, Amazônia

## Abstract

**Introduction:**

Vaccines are very important to reduce morbidity and mortality by preventable infectious diseases, especially during childhood. Optimal coverage is not always achieved, for several reasons. Here we assessed vaccine coverage for the first 12 months of age in children between 12 and 59 months old, residing in the urban area of a small Amazonian city, and factors associated with incomplete vaccination.

**Methods:**

A census was performed in the urban area of Assis Brasil, in the Brazilian Amazon, in January 2010, with mothers of 282 children aged 12 to 59 months old, using structured interviews and data from vaccination cards. Mixed logistic regression was used to determine factors associated with incomplete vaccination schemes.

**Results:**

Only 82.6% of all children had a completed the basic vaccine scheme for the first year of life. Vaccine coverage ranged from 52.7% coverage (oral rotavirus vaccine) to 99.7% coverage (for Bacille Calmette-Guérin). The major deficiencies occurred in doses administered after the first six months of life. Incomplete vaccination was associated with not having enough income to buy a house (aOR = 2.12, 95% CI 1.06-4.21), low maternal schooling (aOR = 2.60, 95% CI 1.28 – 5.29) , and time of residence of the child in the urban area of the city (aOR = 0.73, 95% CI 0.55 – 0.95).

**Conclusions:**

This study showed that vaccine coverage in the first twelve months of life in Assis Brasil is similar to other areas in the Amazon and it is below the coverage postulated by the Brazilian Ministry of Health. Low vaccine coverage was associated with socioeconomic inequities that still prevail in the Brazilian Amazon. Short and long-term strategies must be taken to update child vaccines and increase vaccine coverage in the Amazon.

## Resumo

**Introdução:**

As vacinas são muito importantes para reduzir a morbidade e mortalidade por doenças infecciosas evitáveis, especialmente durante a infância. A cobertura ideal nem sempre é conseguida por várias razões. Aqui nós avaliamos a cobertura vacinal das vacinas referentes ao primeiro ano de vida em crianças entre 12 e 59 meses de idade, residentes na zona urbana de uma pequena cidade da Amazônia, e os fatores associados com a vacinação incompleta.

**Métodos:**

Um censo foi realizado na área urbana de Assis Brasil, na Amazônia brasileira, em janeiro de 2010, com mães de 282 crianças de 12 a 59 meses de idade, por meio de entrevistas estruturadas e dados de cartões de vacinação. O método de regressão logística mista foi utilizada para determinar os fatores associados com esquemas de vacinação incompletos.

**Resultados:**

Apenas 82,6% das crianças completaram o esquema básico de vacina para o primeiro ano de vida. A cobertura vacinal variou entre 52,7% (cobertura para a vacina contra o rotavírus oral) até 99,7% (cobertura da vacina do Bacilo de Calmette-Guérin). As principais deficiências ocorreram em doses administradas após os primeiros seis meses de vida. A vacinação incompleta foi associado com não ter renda suficiente para comprar uma casa (ORa = 2.12, IC 95% 1.06-4.21), baixa escolaridade materna (ORa = 2.60, IC 95% 1.28 – 5.29), e tempo de residência da criança na área urbana da cidade. (ORa = 0.73, IC 95% 0.55 – 0.95).

**Conclusões:**

Este estudo mostrou que a cobertura vacinal dos primeiros doze meses de vida, em Assis Brasil, é semelhante ao de outras áreas na Amazônia e está abaixo da cobertura postulada pelo Ministério da Saúde do Brasil. A baixa cobertura vacinal foi associada com as desigualdades socioeconômicas que ainda prevalecem na Amazônia brasileira. Devem ser tomadas as estratégias de curto e longo prazo para atualizar as vacinas da criança e aumentar a cobertura vacinal na Amazônia.

## Introduction

Child vaccination in the first year of life is important to reduce morbidity and mortality due to infectious diseases [1]. Brazil has launched several Immunization Programs since 1973 [2] and the Ministry of Health (MoH) promotes vaccine campaigns frequently to control and eradicate infectious diseases in children [3]. The evaluation of these programs allows the identification of putative factors that may lead to incomplete vaccination and improper adhesion to the vaccine calendar, thus providing tools for better program management.

In 1973 the Brazilian Government implemented the National Immunization Program (NIP), aiming to control immunization actions over the whole country. The successful implementation of this nationwide program can be attributed to three major events: the fact that smallpox was eradicated in 1973 after a governmental national campaign; the creation of CEME (Central de Medicamentos or Drug Central) in 1971, a governmental organism that was responsible for manufacturing and distributing immune biological products in Brazil; and the Health Decennial Plan for the Americas in 1972, which emphasized the need for coordinated efforts for the control of preventable infectious diseases [4]. Over the last 40 years of existence, the MoH National Immunization Program was able to eradicate smallpox, poliomyelitis and almost achieve measles eradication in Brazil, showing the excellent results of this governmental program [5].

Currently, the NIP has as main targets to maintain the eradication of poliomyelitis; to eradicate hepatitis B, diphtheria, neonatal tetanus, measles, yellow fever, rabies, severe tuberculosis and invasive *Haemophilus influenzae* b disease; and to control other infectious and non-infectious diseases in specific risk groups, including typhoid fever, hepatitis A, human rabies, neonatal transmission of hepatitis B virus, vaccine against varicella zoster virus, and antivenom serums for accidents caused by snakes, spiders and scorpions [2].

To achieve that, the NIP coordinates the distribution and administration of immune biological products in the country, both those that are recommended for the general population, and those targeted to specific groups or diseases, such as immunosuppressed patients, ethnic groups, and occupational immune preventable diseases. All these vaccines are provided free of charge for Brazilian residents and foreigners that are eventually travelling or residing temporarily in the country. This is aimed to guarantee universal access to the vaccines, which usually occurs when people search for vaccines at Health Units during the entire year, or by active campaigns highly publicized in the media, when health workers and volunteers are mobilized to increase access to one or more specific vaccines. The NIP also conducts special operations for target groups, such as remote indigenous tribes in the Amazon.

The NIP has elaborated a basic vaccine calendar for children aged 0 to 12 months old, considering immunological and epidemiological aspects of the targeted diseases, and that calendar includes single dose vaccines such as Bacillus Calmette-Guerin, and multiple doses vaccines, such as Hepatitis B virus vaccine. The composition and recommended age for administration of doses have changed over the years, adapting the calendar to the changes in transmission dynamics for each disease, or the acquisition of new knowledge about some diseases. For instance, new vaccines or new doses were introduced (such as MMR in 1993 and hepatitis B vaccine in 1989); other vaccines were excluded, such as smallpox vaccine in 1980; while changes in the recommendation of some vaccines after severe side-effects and substitution of vaccine formula also happened, as seen with yellow fever vaccine in 1998 and poliomyelitis vaccine in 2012, respectively.

Despite all these government efforts, studies performed in Brazil have shown that vaccine coverage in the first year of life varies according to vaccine type and country region, with an average coverage of 67% for the country [6], which is much below the recommended 90-95% coverage for this age group [7]. Although some Brazilian capitals have reached adequate vaccine coverage within the past years, some of the cities located in the Amazon region still present coverage less than 50% for this age group [6].

Previous studies in Brazil and in other countries have shown that full vaccine coverage may not be reached because of parental cultural and socioeconomic factors [8], zone of residence [9,10], improper access to health care or low quality care [8] or acute diseases that prevent vaccination on scheduled dates [3].

The aim of this study was to assess vaccine coverage in children between 12 to 59 months of age in a small town located in a remote area of the Amazon region, trying to identify factors associated with not completing the vaccine calendar for the first year of life, so vaccine programs can come up with improved strategies to increase such coverage.

## Material and methods

### Study area

Assis Brasil is a small town in the Western Brazilian Amazon and it is located 344 miles southwest of Rio Branco, capital of the state of Acre, Brazil [11]. As of 2010, it had a population of 6,072 inhabitants (3091 men and 2981 women), of which 61% resided in urban areas [12]. The 2010 census counted 770 children aged less than 5 years old living in urban and rural areas, representing 12.68% of the total population of the city.

### Study design and population

The study population included all children between 12 and 59 months old residing in the urban area in January 2010 (307 children) identified using the census records of the only Public Health Unit, who were residing in 252 households.

Data was collected in a cross-sectional design in January 2010, through interviews using structured quantitative questionnaires. Interviewers (FLCCB, TMP, BMD, AMB, HOG, SASM, ACM and CSMO) were trained by MdaSN in November 2009 by applying the same questionnaire used in the study population in a pilot study conducted with a randomly selected sample of 30 mothers at a Health Unit in Rio Branco, were the research team was based. The questionnaire contained questions about family socio-economic status and housing conditions (type of household, monthly income, possession of consumable goods, distance of the house from the health unit, and whether the house was owned or not owned), maternal characteristics (age, schooling degree), gestational characteristics (maternal prenatal care, number of previous pregnancies), child demographic information (birth date, sex and ethnicity); child access to services and care (whether the child had a medical appointment in the previous 12 months, and maternal figure responsible for the child), child morbidities (morbidities in the past 15 days and in the past 12 months, hospital admission), and whether or not the child had lived in rural or riverine areas before 2010. A global positioning system device with precision of 5 meters (Garmin Etrex) was used to locate each house and the distance between household location and the health post was calculated in meters using GPS records. Data on vaccination was collected from children’s health card, which contains official vaccine records. Ethnicity information was collected for both mother and father, who declared their ethnicity. Children from indigenous origin were considered to be those that: (a) had an indigenous surname indicating their indigenous ethnicity, and/or (b) were born from an indigenous mother, indigenous father, or both.

### Basic vaccine calendar

Between 2005 and 2010, the Brazilian vaccine calendar for the first 12 months of life included seven vaccines totalizing 14 doses, which are depicted in Table 1 [2,13].

**Table 1:** **Brazilian vaccine calendar for the first year of life, used between years 2005 and 2010**

**Vaccine**	**Number of doses**	**Age**	**Recommended coverage by MoH**
Bacille Calmet-Guérin	Single	At birth	90%
Hepatitis B	Three	At birth, 30 days and six months of age	95%
Diphtheria-Tetanus- Pertussis-Haemophilus b	Three	At two, four and six months of age	95%
Oral Polio	Three	At two, four and six months of age	95%
Oral Rotavirus^a^	Two	At two and four months of age	90%
Yellow fever	Single	At nine months of age	100%
Measles-Mumps-Rubella	Single	At twelve months of age	95%

The calendar used between 2005 and 2010 included 3 single-dose vaccines and 4 multiple-dose vaccines. BCG was applied intradermal, Hepatitis B, DTP-Hib, Yellow fever and MMR were applied intramuscular, and OPV and OR were given orally. ^a^Oral Rotavirus was included in the calendar in 2006 only. *MoH* = Ministry of Health.

Vaccine shots were analyzed regarding coverage and valid doses. For the coverage study, it was considered to have a complete vaccine scheme those children that received all doses of the vaccines Bacille Calmette Guérin (BCG), Hepatitis B vaccine (HepB), Oral Polio vaccine (OPV), Measles-Mumps-Rubella vaccine (MMR), Diphtheria-tetanus-whole cell pertussis-Haemophilus b (DTP-Hib) and Yellow Fever vaccine (YFV), regardless of when it was taken. Children that did not receive one or more shots of the basic calendar for the first 12 months of life until the date of the interview were considered to have an incomplete vaccination status. By this criteria doses belonging to the first year of life calendar were included in the analysis even if taken after that age.

For a descriptive analysis of valid doses, a vaccine dose was considered to have been taken in proper time if the shot was received up to 7 days after the scheduled date, considering that the proper vaccination date is the age in days indicated by the Brazilian National Immunization Program for each vaccine dose. Although some studies may use up to one month interval period for schedule compliance, we adopted a more strict interval because there are many doses to be administered in the first year of life which are only one or two months apart.

The oral rotavirus vaccine (ORV) was not included in the analysis of complete coverage because it was not in the Brazilian calendar for all the years studied, but it was included in the analysis of valid doses (Table 2) for descriptive purposes only.

**Table 2:** **Prevalence of vaccine coverage, valid doses and length of delay for all doses included in the first year of life, Assis Brasil, 2010**

**Vaccine and dose**	**N**	**Children that took the dose**		**Proper age (in days) for a valid dose**	**Prevalence of valid doses** ^**a,b**^ **n %**		**Average days of delay**	**Minimum days of delay**	**Maximum days of delay**
		**n**	**%**		**N**	**%**			
**BCG**	282	281	99.7	1	202	72.2	25.7	1.5	501
**HepB 1st dose**	281	280	99.6	1	213	76.1	19.5	1	995
**HepB 2nd dose**	281	274	97.5	30	194	70.8	30.6	5	629
**HepB 3rd dose**	281	266	94.7	180	137	51.5	55.2	16.5	838
**DTP-Hib 1st dose**	281	268	95.4	60	158	59.2	32.0	7	934
**DTP-Hib 2nd dose**	281	263	93.6	120	159	60.7	35.5	8	888
**DTP-Hib 3rd dose**	281	256	91.1	180	158	62.0	33.2	7	373
**OPV 1st dose**	281	276	98.2	60	166	60.1	41.3	7	1520
**OPV 2nd dose**	281	271	96.4	120	166	61.3	39.6	8	888
**OPV 3rd dose**	281	260	92.5	180	153	58.8	37.0	9	705
**ORV 1st dose**	221	152	69.1	60	96	63.2	23.2	5	550
**ORV 2nd dose**	221	116	52.7	120	83	71.6	13.4	3	383
**Yellow Fever**	281	266	94.7	270	106	39.8	69.5	16	1028
**MMR**	281	256	91.1	365	99	38.7	68.6	18	854
**Hepatitis B (3 doses)**	281	266	94.7		78	28.2			
**ORV (2 doses)**	221	116	52.7		67	47.2			
**OPV (3 doses)**	281	260	92.5		75	27.9			
**DTP-Hib (3 doses)**	281	256	91.1		78	29.8			
**Complete vaccine status for the first year of life** ^**c**^	282	233	82.6		6	2.3			

The table depicts the number of children that took each vaccine dose, frequency of valid doses (taken until 7 days after the ideal age in days) and age (in days) considered adequate for a valid dose, and time of delay (average, minimum and maximum days of delay). The lower portion of the table shows summarized data for vaccines that require multiple doses (HBV, ORV, OPV and DTP-Hib). The last line show how many children had completed their vaccination for the first year of life, taking into account all doses received until the date of the interview, except for ORV doses. ^a^Valid dose = taken until 7 days after the ideal age in days; ^b^ only doses with a understandable date were computed; ^c^ ORV doses not included.

### Statistical analysis

A household wealth index was created based on the presence of twenty-one consumer goods and household appliances (television, stereo, DVD player, gas stove, refrigerator, washing machine, telephone, bicycle, blender, electric iron, car, sofa, satellite dish, mobile phone, motorcycle, computer, boat, motor boat, water well, power generator and microwave oven) as described in previous publications/studies [11,14,15], using principal component analysis in the XLSTAT software, version 7.5.2 (Addinsoft, New York, NY). The Jolliffe method adapted for the covariance matrix [16] was used to select only variables that contributed to explain most of the variation in the data, and to exclude variables that were not helpful in explaining observed differences, e.g. that where extremely common or extremely uncommon. Only thirteen variables were maintained in the score. The first principal component explained 30.06% of total variance in 2010. The scores for each variable were added to estimate the household wealth index, which was stratified in quartiles.

A database was created with SPSS 13.0 software (SPSS Inc., Chicago, IL) and imported to R software version 2.14.0 (The R Foundation for Statistical Computing). Exploratory univariate logistic regression analysis, using R software, examined potential risk factors and confounders. The dependent variable used was ‘incomplete vaccination status’ (not having received one or more doses belonging to the basic calendar for the first 12 months of life until the date of the interview). Covariates were maintained in subsequent multivariate models, if they were associated with the outcome, at a level of significance of 20%. A stepwise forward method was used to determine variables associated with the outcome using α = 0.05 critical level in adjusted analysis. Model fitness was assessed as described in detail elsewhere [11].

We next used mixed-effects logistic regression (MASS library of R program) to check for the effect of nested data (282 children distributed in 235 households) using the household identification as a random effect in the model, and all other covariates as fixed-effects with the MASS library for R 2.14.0. The final model retained the variables ownership of household, mother schooling and time of residence of the child in the urban area.

### Ethical considerations

The study was approved by the Ethics Committee for Experimentation with Humans at the Federal University of Acre, as required by the Brazilian Ministry of Health, under protocol number 23107.014335/2009-69. We obtained informed written consent from the legal guardian of each participant after the nature and possible consequences of the studies had been fully explained.

## Results

From the 307 children located in the census, 25 children either did not have the official health card, or had incomprehensible written information, and they were excluded from the analysis. These children were similar to those included in the study; except for their ethnicity (most of them were indigenous).

### Analysis of coverage and valid doses

From the 14 vaccine doses programmed to the first 12 months of age, BCG vaccine had the largest coverage (99.70%), followed by HepB vaccine (99.60%). Vaccines that achieved the smaller coverage were the first and second dose of ORV, both below 90%. The highest frequency of shot delays occurred with Yellow Fever vaccine, MMR, and the third dose of HepB vaccine. Vaccine doses that were more frequently received in proper time included BCG, the first and second dose of HepB, and the second dose of ORV (Table 2).

For vaccines with multiple doses, schemes that were more frequently completed were HepB vaccine (94.7%), OPV (92.5%) and DTP-Hib (91.0%), although less than 30% of the children completed them without delay (Table 2). The vaccine ORV had the worst coverage (only 52.7% completed both doses), which can be explained by the fact that the second dose is age-restricted and cannot be applied after 5 months and 15 days of age. (Table 2). About 82.6% of all children received all doses programmed for the first year of life, but only 2.3% received all of the doses in less than 7 days from their recommended ages.

### Factors associated with incomplete vaccination

The main factors associated with not completing the vaccination basic schedule for the first 12 months of life were maternal education, possession of the household, and time of residence outside the urban area.

The population studied had unfavorable socioeconomic conditions, living mostly in wooden houses (Table 3), and having a monthly income lower than US $ 150 per month in 42.2% (n = 112) of the cases. Not owning a house, a very unfavorable socioeconomic condition, was a strong predictor of incomplete vaccination in adjusted analysis (aOR = 2.12, p = 0.033).

**Table 3:** **Socioeconomic characteristics and unadjusted and adjusted odds ratio of having an incomplete vaccine status, Assis Brasil, 2010**

***Socioeconomic variables***	***N (or average*** ^***a***^ ***)***	***Incomplete vaccination***	***Univariated logistic regression***		***Mixed multiple logistic regression*** ^***c***^	
		***n (%)***	***uOR (95%CI)***	***P value*** ^***b***^	***aOR (95% CI)***	***P value*** ^***b***^
**Type of household**						
Brick walls	31	02 (06.45)	1		-	-
Wooden walls or another materials	251	47 (18.73)	3.34 (0.77 – 14.49)	0.107	-	-
***Possession of household***						
Owned	211	29 (13.74)	1		1	
Not owned (rented/borrowed/others)	71	20 (28.17)	2.46 ( 1.29 – 4.71)	0.007	2.12 (1.06 – 4.21)	0.030
***Monthly income***						
<= one Brazilian minimum salary^d^	112	26 (23.22)	1		-	-
> one Brazilian minimum salary^d^	153	22 (14.38)	0.56 (0.30 – 1.04)	0.067	-	-
***Wealth index***						
Richiest half	144	18 (12.50)	1		-	-
Poorest half	138	31 (22.46)	2.03 (1.07 – 3.83)	0.029	-	-
**Household distance from health post (in meters)**	2,995^a^	49 (17.37)	1.0001 (1–1.0002)	0.072	-	-

^a^Average; ^b^Wald test; ^c^Mixed multivariate model included possession of a house, maternal years of schooling and years of residence in urban area; ^d^Brazilian minimum salary in 2010 (R$ 340 = US$ 150). Incomplete vaccine status was defined as not having taken one or more doses required for the first year of life according to the National Immunization Plan adopted between 2005 and 2010. ORV doses were not included in these analysis because they were implemented only after 2006.

About 53.73% of the mothers/caregivers had less than 8 years of education. The odds of not completing the vaccine scheme increased significantly in those mothers with less than 8 years of schooling, when compared with those with more education (aOR = 2.60, p = 0.010), even when adjusting for possession of the household.

Some of the children (n = 17, 6.03%) were either born or have lived in rural/riverine areas of the Amazon before. Living in such areas was significantly associated with incomplete vaccination, while living in urban areas was a protective factor. The average time of residency in the urban area of Assis Brasil was 2.78 years (median = 2.79, range = 0.02 to 4.99), and each year of residence in the urban area provided an additional chance of 29% of completing the vaccine calendar for the first year of life (aOR = 0.71, p = 0.010, Table 4).

**Table 4:** **Child characteristics and unadjusted and adjusted odds ratio of having an incomplete vaccine status, Assis Brasil, 2010**

***Child variables***	***N (or average*** ^***a***^ ***)***	***Incomplete vaccination***	***Univariated logistic regression***		***Mixed multiple logistic regression*** ^***c***^	
		***n (%)***	***uOR (95%CI)***	***P value*** ^***b***^	***aOR (95% CI)***	***P value*** ^***b***^
**Gender**						
Male	139	18 (12.95)	1		-	-
Female	143	32 (22.38)	1.86 (0.99 – 3.51)	0.043	-	-
***Age***	2.98^b^	49 (17.37)	0.84 (0.65 – 1.09)	0.198	-	-
**Maternal figure**						
Biological mother	259	46 (17.76)	1		-	-
Other than biological mother	23	3 (13.04)	0.69 (0.20 – 2.44)	0.569	-	-
**Has the child lived in rural or riverine areas before?**						
No	265	42 (15.85)	1		-	-
Yes	17	7 (41.18)	3.72 (1.34 – 10.31)	0.012	-	-
**Time of residency in the urban area (in years)**	2.78^b^	49 (17.37)	0.71 (0.55 – 0.92)	0.010	0.73 (0.55 – 0.95)	0.028
**Has the child had a health care consult in the previous 12 months?**						
No	26	7 (26.92)	1		-	-
Yes. when sick only	170	28 (16.47)	0.54 (0.21 – 1.39)	0.200	-	-
Yes. once a while	48	9 (18.75)	0.63 (0.20 – 1.94)	0.417	-	-
Yes. frequently	34	4 (11.76)	0.36 (0.09 – 1.40)	0.142	-	-
**Has the child ever been admitted to hospital?**						
No	159	32 (20.13)	1		-	-
Yes	122	17 (13.93)	0.64 (0.34 – 1.22)	0.177	-	-

^a^Average; ^b^Wald test; ^c^Mixed multivariate model included possession of a house, maternal years of schooling and years of residence in urban area.

The distance between the location of the houses and the health post varied from 281.10 to 15,940 meters. It was associated with incomplete vaccination during the initial steps of the multiple logistic regression, but when adjusted by maternal education this association disappeared.

Maternal access to prenatal care was high (n = 237, 95.56%), and the number of consults for those mothers that attended prenatal care ranged from 2 to 17 (average 13.40 consults). The number of lifetime pregnancies was in average 3.45 (range = 1 to 15). Each additional pregnancy increased the chance of not completing the vaccine scheme by 15% (p = 0.018), but when adjusted by socioeconomic variables this association disappeared, showing that pregnancies were related to socioeconomic conditions. Neither the access nor the number of prenatal care consults had association with vaccine status either (Table 5).

**Table 5:** **Maternal characteristics and unadjusted and adjusted odds ratio of having an incomplete vaccine status, Assis Brasil, 2010**

***Maternal variables***	***N (or average*** ^***a***^ ***)***	***Incomplete vaccination***	***Univariated logistic regression***		***Mixed multiple logistic regression*** ^***c***^	
		***n (%)***	***uOR (95%CI)***	***P value*** ^***b***^	***aOR (95% CI)***	***P value*** ^***b***^
**Age (in years)**	29.32^**a**^	49 (17.37)	0.98 (0.94 – 1.02)	0.279	-	-
**Years of schooling**						
8 and more	130	13 (10.00)	1		1	
0 to 8 years	151	35 (23.18)	2.72 (1.37 – 5.39)	0.004	2.60 (1.28 – 5.29)	0.011
***Ethnicity***						
Non-indigenous	253	39 (15.42)	1		-	-
Indigenous	25	8 (32.00)	2.58 (1.04 – 6.40)	0.040	-	-
***Attended prenatal care***						
No	11	3 (27.27)	1		-	-
Yes	237	41 (17.29)	0.56 (0.14 – 2.19)	0.403	-	-
***Number of prenatal care consults***	13.40^a^	49 (17.37)	0.93 (0.84 – 1.03)	0.184	-	-
***Number of current pregnancies***	3.45^a^	49 (17.37)	1.15 (1.02 – 1.29)	0.018	-	-

^a^Average; ^b^Wald test; ^c^Mixed multivariate model included possession of a house, maternal years of schooling and years of residence in urban area. Incomplete vaccine status was defined as not having taken one or more doses required for the first year of life according to the National Immunization Plan adopted between 2005 and 2010. ORV doses were not included in these analysis because they were implemented only after 2006.

Two other variables were associated with not completing the vaccination in the unadjusted analysis, but when adjusted by socioeconomic variables or maternal education no statistical significance was found. They included maternal/caregiver ethnicity (Table 5) and the socioeconomic index based in ownership of consumable goods (Table 3).

Maternal age (Table 5), child sex and age, and maternal figure in care of the child did not show association with incomplete vaccination (Table 4). Having access to health care or being hospitalized before did not have association with incomplete vaccination either (Table 4).

## Discussion

The overall vaccine coverage for the first 12 months of age in Assis Brasil (82.60%) is very close to vaccine coverage in the capital of the state (79.20%) and in other large Amazonian cities, which ranged from 62.1% in Macapá to 84.50% in Boa Vista [6]. The reported coverage prevalence in Assis Brasil is also identical to the national average coverage of 82.60%, in a study that was conducted with 17,295 Brazilian Children in all (27) capitals [6]. Therefore, the results obtained here can be considered representative of our population and comparable to the general Brazilian population. The recommended vaccine coverage by MoH varies between 90% (for BCG and Rotavirus) to 95% (all other vaccines), and according to Barata *et al*. [6] only a few capitals in Brazil have achieved it, mainly in the Southern part of Brazil (Porto Alegre, Florianopolis, Curitiba), and in Brasilia, Cuiaba and Teresina (none of which are located in the Amazon).

When considering each vaccine separately, the only vaccine doses in children from Assis Brasil that reached the ideal coverage recommended by the Brazilian MoH (90% for BCG and OPV, 100% for Yellow Fever and 95% for all others) were the first and second doses of OPV, the first and second doses of HepB, the first dose of DTP-Hib and BCG. All other vaccine doses were close to recommended coverage (between 91% and 94.7%), except for the vaccine ORV.

In the Southern region of Brazil, where vaccine coverage is the highest in the country [6], two studies [17,18] also showed that BCG, DTP-Hib, OPV and HepB were the only vaccines that reached the ideal coverage proposed by the Brazilian MoH. On the other hand, vaccines with the lowest coverage in South Brazil (Yellow Fever, with 85.70% coverage, and MMR with 85.8%) also had similar lower coverage in Assis Brasil, showing that these studies performed in different regions in Brazil are comparable and that the problem is related to the age the dose should be given. Vaccine doses that are programmed for late childhood are more prone to not be taken, or to be administered with a large amount of delay.

In our study, vaccine schemes composed of more than one dose were more likely to be delayed, and less than 30% of the children received HepB vaccine, DTP-Hib and OPV in proper time. Similar results were found in other areas in the Amazon [19] and also in Southern Brazil [17], as well as in other countries such as India and Nigeria [20]. Therefore, health units must develop strategies to encourage parents to take their children to the health unit in order to take their shots regularly, and at the same time increase the search for children with already incomplete vaccine schemes [17,19].

The vaccine ORV was implemented in Brazil in March 2006, and a very low coverage was observed in our study for both doses for children born after that date. The Brazilian Ministry of Health reported a national coverage of 82% for this vaccine in 2009, while WHO reported 93% coverage for Brazil in 2010 [21]. The same WHO report indicated that only six other countries in the American continent reached more than 90% coverage for this vaccine (Ecuador, El Salvador, Honduras, Mexico, Nicaragua and Panama), Therefore Assis Brasil had an extremely lower coverage when compared to the national Brazilian average [22], probably indicating a local problem with this vaccine. It is possible that it took a while to distribute this vaccine in the Amazon and in other countries in Latin America, what could explain these results [19].

WHO report for vaccine coverage for the first year of life varies according region of the World. In the Americas, BCG is the vaccine with the highest number of countries with at least 90% coverage (84.00%). The frequency of countries in Latin America which achieved coverages of at least 95% of children less than 1 year old was 53.48% for DTP, 56.09% for HepB, 55.81% for MMR and 48.83% for VOP [21]. This data shows that the Brazilian NIP is very well succeeded when compared to other countries in Latin America.

Vaccine coverage in other areas of the world also varies a lot. Data from mid 2000’s shows that in some African countries, vaccine coverage in children less than 5 years old does not reach 80.00% of coverage (Ethiopia, Nigeria and Comoros), while others are achieving more than 95.00% [23]. In India, vaccine coverage was between 90 and 95% in 2006, and in Asia, reported vaccine coverage under 5 years old also varies between 91 and 99% [23].

The WHO estimates vaccine coverage worldwide as 84% for DTP. Coverages over 90% for DTP3 are obtained in the Americas, Europe and Western Pacific only. Hepatitis B vaccine has an estimated coverage of 81% worldwide, being higher in the Western Pacific (92%), but very low in Africa (11%). Vaccine coverage for Polio is estimated to be 84%, and 3 countries were still considered polio-endemic areas in 2013. Rotavirus vaccine has recent introduction in many countries, and overall coverage in 2013 was estimated to be 14%. For yellow fever vaccine, coverage in 44 countries and territories at risk is estimated to be only 41%. *Haemophilus influenzae* type B vaccine coverage with three doses is estimated to be 52% worldwide, being higher in the Americas (90%), but low in the Western Pacific Region (only 18%). Measles has a worldwide coverage of 84% [24].

Although some studies performed in other countries, such as Kenya [9] have shown an association between the possession of consumable goods and vaccine status, in our study this wealth indicator did not remain associated with vaccine status after adjustment by maternal schooling, maybe because it is a short-term indicator of wealth, and children in the study were between 12 and 59 months old. Barata et al. [6] did not find association with wealth index and incomplete vaccination after adjustment by maternal schooling in 27 Brazilian capitals either. The best wealth indicator which was independently associated with vaccine status in Assis Brasil was possession of a household, probably because it is a long-term wealth indicator, since the acquisition or building of a house requires more stable financial conditions.

Konstantyner et al. [25] has demonstrated that inadequate housing conditions related to low income were associated with incomplete vaccine status in children in Brazil. Queiroz et al. [26] has also shown higher inadequate vaccine coverage in children belonging to low income families. It is possible that parental low socioeconomic conditions may result in less time available for parental care, because mothers and other members of the family have to work in order to sustain the household. In this case, it would be more difficult to vaccinate children during Health Unit working hours (which are usually during daylight), since lost working hours could result in salary deductions for these mothers. In fact, [27] have shown that mothers that were not able to take maternity leave were less likely to have an updated vaccination card for their children, showing the interference of work into the ability to follow vaccination recommendations. On the other hand, for those living away from the Health Unit, the cost of taking the child to the vaccination service could be a barrier to some low-income families, even when not having to pay for the vaccine, as it is the case in Brazil. The third possible explanation is that in many settings low family income is associated with low education, either from the mother, the chief of the household, or both.

Studies performed in the nineties [28,23,29-31] have shown an association between maternal educational level and incomplete vaccine status in their children, because these mothers had difficulty in understanding the importance of vaccines in preventing diseases. Ten years later, this association disappeared in Brazilian areas where some socioeconomic development was achieved [32,6,33], probably because it contributed to higher access to information about vaccines and disease prevention. It may also have improved wealth status in groups of lower schooling. Barros et al. [34] have shown that maternal education is changing over the years in socioeconomic quintiles: while there used to be a correlation between low education and low income two decades ago, now the scenario is changing to a situation where almost 30% of the mothers in the poorest wealth quintile have more than 8 years of education. In our study, low maternal schooling status continued to be an independent predictor of incomplete vaccination even after adjustment by possible confounding factors, showing that parental schooling is still an important predictor of child health in Amazonian areas of lower socioeconomic development.

Several other studies have shown association between maternal education and child health and vaccination status in developing [35-38] and developed countries [39], pointing out maternal education as the major determinant of child health and nutrition [34]. Thus, a major keystone in improving child vaccine coverage is to increase levels of maternal education, since it is the mother (or caregiver) the main responsible for taking the child to the health unit in order to get vaccine. While this requires long-term interventions in many countries, a more feasible short-term strategy is to increase educational campaigns in the media and in schools to bring to the attention of mothers with low levels of education the importance of proper vaccination. At the same time, facilitating access to the vaccines by offering a broader scheduling hours for vaccination at health units for mothers that work on a daily basis during Health Units operating hours may overcome other obstacles for vaccination that accompanies low maternal education.

Direct access to health care can influence vaccine compliance. The relationship between place of residence and location of health care services has been evaluated in some studies [40,41], showing that the higher the distance between the house and the health post, the less likely children would get vaccinated. In Assis Brasil, children living in houses that were distant from the health post had less chance to get their vaccines, but this association disappeared after adjusting by maternal schooling and wealth indicators, suggesting that low income mothers or mothers with low schooling had to live in the most distant houses and it was these conditions that prevented them from vaccinating their children rather than the physical distance from the health post. At the same time, maternal access to prenatal care or child access to medical care and hospitalization were not associated with better vaccine coverage, as shown in other studies [9,10]. It is possible that the health care workers are not using properly every single family visit to Health clinics to orient about the importance of vaccines, or to update the child’s vaccination card.

In our study, living in rural areas of the Amazon increased the odds of not completing the vaccine scheme for the first year of life, and each year of residence in the urban area of the city decreased the probability of having incomplete vaccine status by 29%. This result exemplifies the difficulties in accessing vaccine services outside urban areas in the Amazon. Family health programs located in the Amazon must pay attention to this particularity of health care access in children coming from rural and riverine areas. Other researchers have also detected difficulties in vaccine access in rural areas, together with other social inequities [9,10].

Vaccines are important to child health, since they can prevent morbidity and mortality by infectious diseases. Therefore, adherence to the vaccine calendar is a keystone in child health. Strategies for child health improvement have to target increased vaccine coverage and compliance to the calendar, not delaying scheduled doses. Vaccines will help prevent some diseases and reduce morbidity, but other measurements have to be taken, such as increasing food access and food security for children, promoting breastfeeding, reducing undernutrition and preventing overweight and obesity, and most of all, reducing child mortality. Although vaccination is important to improve child health, reducing social inequities is also important to decrease exposure to disease determinants, such as poor sanitation, contaminated environments, poor quality water, food insecurity and lack of understanding of basic disease determinants. Brazil has been acting on trying to improve child health with the implementation of the National Immunization Program since 1973, and several child and maternal health programs since the 80’s [34,42].

In 2009, the Brazilian Government launched the Pact for Reduction of Child Mortality in the Northeast and the Amazon, aiming to reduce child mortality by 5% in these two regions in the years 2009 and 2010, targeting preventive measures such as prenatal and postnatal care, breastfeeding and increased capacity of the health system to treat severe cases of respiratory diseases, dehydration and maternal intercurrences, and strengthening the Family Health teams, that are responsible for overviewing the child vaccination program. However, this Pact was partially successful, because child mortality in the Amazon decreased from 23.10% to 21% only [43].

The reasons why plans such this and others are not highly effective is that they face specific challenges for areas such as the Amazon. These challenges include areas of difficult and remote access with journeys taking several hours, lack of qualified human resources, lack of electricity and vaccine deterioration, low educational level of the most remote population, and frequent migration of children and their families between rural and urban areas. Programs aimed to increase education level of the general population have to be effective in order to result in a more qualified human task force, that can promote the Ministry of Health plans and actions more efficiently. Long-term economic investments are also needed to provide better environmental conditions, such as 24-hour electricity availability in the Health Units, among other possible strategies.

The importance of this study is that it was a population-based study and collected information directly from the vaccine card, therefore eliminating recall bias that exists using only parent’s report. However, it also has some limitations. The first one is that incomplete vaccination may have been underestimated, because children not having an official card (which probably reflects less parental concern about health), were excluded from the analysis. The second limitation is that most of the excluded children were of indigenous origin and therefore the power of the study in detecting association between ethnicity and lack of immunization was reduced. Studies performed in other areas of Brazil disagree about the role of ethnicity in achieving optimal vaccine status. While some studies did not find association between ethnicity and vaccination [1,6], a study performed in Northeast Brazil [32] found that children of black origin are less likely to get proper vaccination after controlling for socioeconomic confounding factors, possibly because of racial discrimination, as explained by these authors. Since the Amazon region has a special composition regarding ethnicity, with a high frequency of indigenous people, more specific studies should be performed in this region before reaching a conclusion. The third limitation is that the study was not directed to evaluate factors associated with delayed vaccination, which could have detect other associations with vaccine compliance reported in the literature, such as previous and recent morbidities [31], number of maternal pregnancies and live siblings [9,29,31], attending prenatal care [10], and type of family member participating in the process of decision-make about the child [23].

## Conclusions

This study showed that vaccine coverage in the first twelve months of life in Assis Brasil is similar to other areas in the Amazon and it is below the recommended coverage of 90%-100% by the Brazilian Ministry of Health, despite the fact that vaccine is offered for free in public health units. The major deficiencies occurred in doses administered after the first six months of life (such as MMR and Yellow Fever vaccines), and ORV, recently introduced in the Amazon by the time this study was performed. Low vaccine coverage was associated with poor family wealth status, less than 8 years of maternal schooling and smaller time of residence in the urban area of the city, being related to socioeconomic inequities that still prevail in the Brazilian Amazon. Short-term measures must be adopted to increase vaccine coverage in risk groups, searching for children already with delayed doses, especially migrants from non-urban areas and children from low income families. Long-term strategies should include investments in public policies that will be able to diminish social inequities, such as education, formal available jobs and vaccine access in rural and riverine areas of the Amazon.
